# Critically ill severe hypothyroidism: a retrospective multicenter cohort study

**DOI:** 10.1186/s13613-023-01112-1

**Published:** 2023-03-09

**Authors:** Simon Bourcier, Maxime Coutrot, Alexis Ferré, Nicolas Van Grunderbeeck, Julien Charpentier, Sami Hraiech, Elie Azoulay, Saad Nseir, Nadia Aissaoui, Jonathan Messika, Pierre Fillatre, Romain Persichini, Serge Carreira, Alexandre Lautrette, Clément Delmas, Nicolas Terzi, Bruno Mégarbane, Jean-Baptiste Lascarrou, Keyvan Razazi, Xavier Repessé, Claire Pichereau, Damien Contou, Aurélien Frérou, François Barbier, Stephan Ehrmann, Etienne de Montmollin, Benjamin Sztrymf, Elise Morawiec, Naïke Bigé, Danielle Reuter, David Schnell, Olivier Ellrodt, Jean Dellamonica, Alain Combes, Matthieu Schmidt

**Affiliations:** 1 Medical Intensive Care Unit, Assistance Publique-Hôpitaux de Paris (APHP), Pitié–Salpêtrière Hospital, 75651 Paris Cedex 13, France; 2 Intensive Care Unit, Versailles Hospital, Le Chesnay, France; 3 Department of Critical Care Medicine, Schaffner Hospital, Lens, France; 4 Medical Intensive Care Unit, Cochin Hospital, Hôpitaux Universitaire Paris Centre, APHP, Paris, France; 5 Réanimation des Détresses Respiratoires et des Infections Sévères, Assistance Publique, Hôpitaux de Marseille, Hôpital Nord, Marseille, France; 6 Medical Intensive Care Unit, Saint-Louis Hospital, APHP, Paris, France; 7 Médecine Intensive-Réanimation, CHU de Lille, 59000 Lille, France; 8 INSERM U1285, Université de Lille, CNRS, UMR 8576 - UGSF - Unité de Glycobiologie Structurale et Fonctionnelle, 59000 Lille, France; 9 Department of Critical Care Unit, Hôpital Européen Georges-Pompidou (HEGP), APHP, Paris, France; 10 Medico-Surgical Intensive Care Unit, APHP. Nord-Université Paris Cité, Hôpital Louis Mourier, 92700 Colombes, France; 11 Medical–Surgical Intensive Care Unit, CH de Saint-Brieuc, Saint-Brieuc, France; 12 Medical–Surgical Intensive Care Unit, Centre Hospitalier Universitaire (CHU) de La Réunion, Felix-Guyon Hospital, Saint-Denis, La Réunion France; 13 Medical–Surgical Intensive Care Unit, Saint-Camille Hospital, Bry-sur-Marne, France; 14 Medical Intensive Care Unit, CHU Clermont-Ferrand, Clermont-Ferrand, France; 15 Intensive Cardiac Care Unit, Cardiology Department, Rangueil University Hospital, 1 Avenue Jean Poulhes, 31059 Toulouse, France; 16 REICATRA, Institut Saint Jacques, CHU de Toulouse, Toulouse, France; 17 Department of Medical Intensive Care, CHU de Grenoble Alpes, Grenoble, France; 18 Department of Medical Intensive Care, Lariboisière Hospital, APHP, Paris, France; 19 Médecine Intensive Réanimation, CHU de Nantes, Nantes, France; 20 Service de Médecine Intensive Réanimation, AP-HP, CHU Henri Mondor, DHU A-TVB, Créteil, France; 21 Intensive Care Unit, University Hospital Ambroise-Paré, APHP, Boulogne-Billancourt, France; 22 Intensive Care Unit, Poissy Saint-Germain-en-Laye Hospital, Poissy, France; 23 Service de Réanimation Polyvalente, Centre Hospitalier Victor Dupouy, Argenteuil, France; 24 Medical Intensive Care Unit, Hôpital Pontchaillou, CHU de Rennes, Rennes, France; 25 Medical Intensive Care Unit, CH Regional d’Orléans, Orléans, France; 26 Médecine Intensive Réanimation, CHRU Tours, CIC INSERM 1415, CRICS-TriggerSep F-CRIN Research Network, Tours, France; 27 INSERM, Centre d’étude Des Pathologies Respiratoires, U1100 Tours, France; 28 Université de Tours, Tours, France; 29 Medical–Surgical Intensive Care Unit, Delafontaine Hospital, Saint-Denis, France; 30 Service de Réanimation Polyvalente et Surveillance Continue, AP-HP, Hôpital Antoine Béclère, 157 rue de la porte de Triveaux, 92140 Clamart, France; 31 Service de Pneumologie et Réanimation Médicale (Département “R3S”), Hôpital de la Pitié–Salpêtrière, APHP, Paris, France; 32 Medical Intensive Care Unit, Hôpital Saint-Antoine, APHP, Paris, France; 33 Medical–Surgical Intensive Care Unit, CH Sud Francilien, Corbeil, France; 34 Service de Réanimation Polyvalente, CH d’Angoulême, Angoulême, France; 35 Département de Médecine Intensive, Groupe Hospitalier Sud Île-de-France, Hôpital de Melun, Melun, France; 36 Service de Médecine Intensive Réanimation, Hôpital Archet 1, Centre Hospitalier Universitaire de Nice, UR2CA Université Cote d’Azur, Nice, France; 37 Medecine Intensive Reanimation, Institute of Cardiometabolism and Nutrition, Sorbonne Universités, INSERM, UMRS_1166-ICAN, Hôpital de la Pitié–Salpêtrière, 47, bd de l’Hôpital, 75651 Paris Cedex 13, France

**Keywords:** Hypothyroidism, Myxedema, Coma, Cardiogenic shock, Critical care

## Abstract

**Background:**

Severe hypothyroidism (SH) is a rare but life-threatening endocrine emergency. Only a few data are available on its management and outcomes of the most severe forms requiring ICU admission. We aimed to describe the clinical manifestations, management, and in-ICU and 6-month survival rates of these patients.

**Methods:**

We conducted a retrospective, multicenter study over 18 years in 32 French ICUs. The local medical records of patients from each participating ICU were screened using the International Classification of Disease 10th revision. Inclusion criteria were the presence of biological hypothyroidism associated with at least one cardinal sign among alteration of consciousness, hypothermia and circulatory failure, and at least one SH-related organ failure.

**Results:**

Eighty-two patients were included in the study. Thyroiditis and thyroidectomy represented the main SH etiologies (29% and 19%, respectively), while hypothyroidism was unknown in 44 patients (54%) before ICU admission. The most frequent SH triggers were levothyroxine discontinuation (28%), sepsis (15%), and amiodarone-related hypothyroidism (11%). Clinical presentations included hypothermia (66%), hemodynamic failure (57%), and coma (52%). In-ICU and 6-month mortality rates were 26% and 39%, respectively. Multivariable analyses retained age > 70 years [odds ratio OR 6.01 (1.75–24.1)] Sequential Organ-Failure Assessment score cardiovascular component ≥ 2 [OR 11.1 (2.47–84.2)] and ventilation component ≥ 2 [OR 4.52 (1.27–18.6)] as being independently associated with in-ICU mortality.

**Conclusions:**

SH is a rare life-threatening emergency with various clinical presentations. Hemodynamic and respiratory failures are strongly associated with worse outcomes. The very high mortality prompts early diagnosis and rapid levothyroxine administration with close cardiac and hemodynamic monitoring.

**Supplementary Information:**

The online version contains supplementary material available at 10.1186/s13613-023-01112-1.

## Introduction

Hypothyroidism is a pathological condition related to a deficiency in circulating thyroid-hormone concentrations [1]. Etiologies are mainly autoimmune thyroiditis, iodine deficiency, post-thyroidectomy or pharmacological, and central due to pituitary or hypothalamic disorders [1, 2]. Although the prevalence of overt hypothyroidism is estimated at 1–2% of the general population [3], hypothyroidism may result in a wide range of severity from subclinical hypothyroidism to exceptional life-threatening myxedema coma (MC).

The term myxedema was first used by Ord in 1877 to describe a severe disorder associated with dry skin, swelling, profound hypothermia, and neurocognitive impairment among other clinical features in previously healthy women. A link was made with cretinism description and symptoms observed after thyroidectomy [4, 5]. Since then, the exact prevalence worldwide remains unknown and actual knowledge of severe hypothyroidism (SH) mainly relies on case reports and small series [6–13]. Through these previous observations, SH can have a large variety of presentations including mild to severe coma, seizures, hypothermia, bradycardia, heart failure, and pericardial effusion leading to multiorgan failure and death. Therefore, clinical diagnosis of SH requires a low index of suspicion and should be further confirmed by investigations of thyroid-stimulating hormone (TSH) and free thyroxine (FT4) [1].

Prognosis has been markedly improved by thyroid hormone replacement therapy, with in-hospital mortality reaching 30–40% in the most recent series [9–13]. Indeed, levothyroxine represents the mainstay of the therapeutic management of SH. Despite poor evidence, the current American Thyroid Association (ATA) guidelines support the intravenous administration of a levothyroxine loading dose and empiric glucocorticoid as part of the initial therapy [14]. Ultimately, SH may lead patients to ICU for organ support and specific management. To date, data on these severe patients admitted to ICU are limited. Herein, we report clinical characteristics, management, and outcomes of a large cohort of SH patients treated in French ICUs.

## Materials and methods

### Study population

This study retrospectively included SH patients hospitalized in 32 ICUs between 2000 and 2017 (Additional file 1: Fig. S1). All consecutive patients with at least one of the following International Classification of Diseases 10 diagnoses E03.5 (Myxedema coma) and E03.9 (Hypothyroidism, unspecified) were screened by the local investigator in each participating ICU. Then, ICU reports were anonymously sent to two investigators (SB and MC), who independently selected patients satisfying inclusion criteria. Disagreements were resolved by consensus after discussion with a third investigator (MS). Inclusion criteria were: adults > 18 years admitted to the ICU with at least one organ failure and/or a Sequential Organ-Failure Assessment (SOFA) score ≥ 1 [15], and SH diagnosis defined by a combination of the following criteria 1, 2, 3, and/or 4: (1) TSH concentration above the upper reference range and/or FT4 concentration below the lower reference range; (2) one or more central nervous system manifestation(s) (somnolence/coma, Glasgow ≤ 14, seizures); (3) hypothermia ≤ 35 °C; and (4) acute circulatory failure (SOFA cardiovascular component ≥ 2).

Patients were excluded when hypothyroidism was not the primary reason for ICU admission and if another etiology could explain the presence of criteria 2, 3, and 4, even when criterion 1 was met.

### Data collection

Baseline information at ICU admission included: demographic data, modified Charlson [16], Simplified Acute Physiology Score II (SAPS II) [17], SOFA score, underlying thyroid disease, triggering factors, clinical signs, and laboratory findings. Follow-up parameters recorded were the use of vasopressors, inotropic drugs, invasive mechanical ventilation, and renal replacement therapy. In addition, the composite diagnosis score of myxedema coma proposed by Popovenic et al. [18] which combines the presence or absence of alterations of thermoregulatory, central nervous systems, cardiovascular, gastrointestinal, and metabolic systems, and the presence or absence of a precipitating event, was applied in our cohort. Specific thyroid management (thyroid hormone replacement therapy) and corticosteroid use in ICU were also reported. Finally, survival at ICU discharge and 6-month survival status after ICU admission (through medical charts or contact by phone) was noted.

### Ethical considerations

This study was approved by the Ethics Committee of the French Intensive Care Society (Société de Réanimation de Langue Francaise CE #17-26) and complied with French research Reference Methodology MR003 regarding health-data privacy, and the French National Commission on Informatics and Liberty (CNIL)*.*

### Statistical analyses

This study followed the STROBE statement recommendations for cohort studies. Continuous variables [expressed as median (interquartile range)] were compared with Student’s *t* test or the Wilcoxon test, as appropriate. Categorical variables [expressed as number (%)] were compared with χ^2^ tests. Patients’ demographic, clinical, management characteristics and laboratory results were tested in bivariate analyses for association with in-ICU mortality and the presence of circulatory failure. Thereafter, factors achieving *p* ≤ 0.10 in bivariate analyses were entered into logistic regression models to investigate variables associated with ICU and 6-month mortality. Logistic regression analyses using backward-stepwise variable elimination were run (with the variable exit threshold set at p > 0.05). Multiple backward-stepwise logistic-regression analyses were used to select the final regression model using the Akaike information criterion. Multicollinearity was assessed by calculating a variance inflation factor of each variable and was ruled out if the variance inflation was lower than 4 and > 0.2. Variables associated with one another were not included in the model. No assumptions were made for missing data (Additional file 1: Table S1). Finally, Kaplan–Meier survival curves were computed according to age (binary), cardiovascular and ventilation components of the SOFA score, and compared with Mantel–Cox log-rank tests. Statistical significance was defined as a p-value of less than 0.05. Analyzes were computed with R 4.0.1 (R Foundation for Statistical Computing, Vienna, Austria) software.

## Results

### Study population

During the 18-year study period, 447 adult patients with at least one organ failure and TSH and/or FT4 concentration outside the reference range were admitted to 32 ICUs. Among this population, SH diagnosis was confirmed for 82 of them (incidence of 5.6 per 100,000 patients admitted, Additional file 1: Fig. S1). Their main characteristics are reported in Table 1. Briefly, they were 70 (59–78) years of age, 74% female, and a SAPS II of 55 (45–70). The main causes of hypothyroidism were thyroiditis (29%) and thyroidectomy (19%), while no evident cause was defined in nearly one-quarter of patients. Besides, a de novo diagnosis of hypothyroidism was made in 44 (54%) patients during their ICU stay. The most frequent SH triggers were levothyroxine discontinuation (28%), amiodarone hypothyroidism (11%), and sepsis (15%). Thirty-three patients (40%) were admitted to the hospital during the winter season (i.e., December, 21 to March, 20 in France).

**Table 1:** Included patients’ general characteristics according to ICU survival status

Characteristic	Total	ICU survivors	ICU nonsurvivors	*p*
	(n = 82)	(n = 61)	(n = 21)	
At ICU admission				
Age (years)	70 (59–78)	68 (58–76)	77 (70–81)	0.015
Female	61 (74)	47 (77)	14 (67)	0.391
SAPS II	55 (45–70)	53 (42–66)	61 (55–88)	0.005
Charlson score	3 (1–5)	3 (1–4)	4 (3–7)	0.013
SOFA score	8 (6–12)	7 (5–11)	12 (8–13)	0.001
Cardiovascular	3 (0–4)	1 (0–4)	4 (4–4)	< 0.001
Respiratory	1 (0–2)	1 (0–2)	2 (1–3)	0.001
Neurological	3 (2–4)	3 (2–4)	4 (1–4)	0.705
De novo hypothyroidism	44 (54)	32 (52)	12 (57)	0.802
Etiology				0.883
Primary	76 (93)	56 (92)	20 (95)	
Thyroidectomy	16 (19)	13 (21)	3 (14)	
Thyroiditis	24 (29)	16 (26)	8 (38)	
Graves’ disease	1 (1)	1 (2)		
Atrophic thyroiditis	1 (1)	1 (2)		
Congenital	1 (1)	1 (2)		
Unknown	20 (24)	16 (26)	4 (19)	
Central	6 (7)	5 (8)	1 (5)	
Trigger				0.726
Levothyroxine discontinuation	23 (28)	17 (28)	6 (29)	
Sepsis	12 (15)	9 (15)	3 (14)	
Amiodarone	9 (11)	5 (8)	4 (19)	
Drug-induced hypothyroidism*	5 (6)	4 (7)	1 (5)	
Unknown	33 (40)	26 (43)	7 (33)	
Diagnostic score for myxedema coma^a^	65 (50–75)	65 (50–75)	70 (50–75)	0.63
TSH (mIU/L)	51.0 (17.5–94.5)	44.0 (17.2–90.0)	63.0 (29.8–100.0)	0.281
FT3 (pmol/L)	1.3 (0.0–2.2)	1.3 (0.5–2.1)	0.1 (0.0–2.2)	0.451
FT4 (pmol/L)	2.7 (0.0–7.5)	3.0 (0.2–7.8)	0.9 (0.0–4.9)	0.247

Results are expressed as median (interquartile range) or *n* (%)

*SAPS II* Simplified Acute Physiology Score II, *ICU* intensive care unit, *SOFA* Sequential Organ-Failure Assessment, *TSH* thyroid-stimulating hormone, *FT3* free triiodothyronine, *FT4* free thyroxine

* Drug-induced hypothyroidism included 1 case secondary to an immune checkpoint inhibitor and 1 secondary to a tyrosine kinase inhibitor

^a^According to Popoveniuc et al., the previously proposed diagnostic scoring system for myxedema coma include a composite of alterations of thermoregulatory, central nervous, cardiovascular, gastrointestinal, and metabolic systems, and presence or absence of a precipitating event

**Fig. 1:**
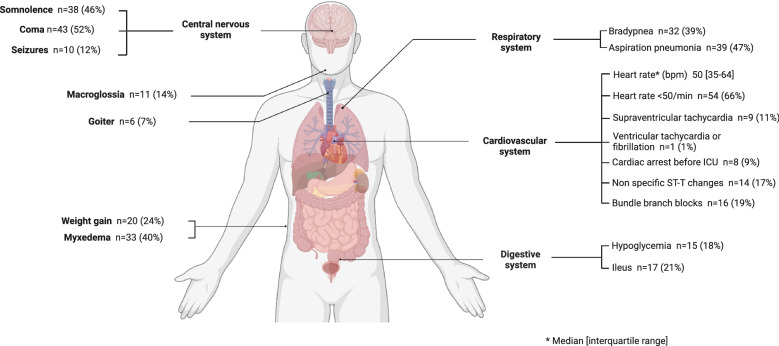
Main clinical presentation of severe hypothyroidism in critically-ill patients

Interestingly, SH clinical findings at ICU admission included multiple systemic manifestations (Fig. 1). First, central nervous system involvement such as Glasgow coma scale < 9 was reported in 43 patients (52%), whereas somnolence (i.e., 9 ≤ Glasgow coma scale ≤ 14) and seizures were reported in 38 (46%) and 10 (12%) patients, respectively. Hypothermia (≤ 35 °C) was frequently observed in 54 (66%) patients, with an in-ICU median lowest temperature of 34.1 °C [31.1–35.9]. Other noticeable features were aspiration pneumonia in 39 (48%), bradypnea in 32 (39%), ileus in 17 patients (21%), and hypoglycemia in 15 (18%) patients. Importantly, hemodynamic impairment, defined as SOFA cardiovascular component ≥ 2, was reported in 47 (57%) patients, whereas bradycardia (≤ 50 beats/min) and cardiac arrest at ICU admission were reported in 41 (50%) and 8 (10%) patients, respectively. The composite diagnosis score of myxedema coma was 65 (50–75) and classifying the population as being highly suggestive of myxedema coma [18]. However, it was not significantly different between ICU survivors and nonsurvivors (Table 1).

Patients with cardiovascular SOFA ≥ 2 had a lower heart rate before ICU admission [40 (30–63) versus 50 (45–64) beats/min, p = 0.05], higher arterial lactate [2.2 (1.5–4.1) versus 1.2 (0.9–2.5) mmol/L, p = 0.014] and were more likely to have cardiac arrest and aspiration pneumonia before ICU admission than those without hemodynamic impairment. On the other hand, patients without cardiovascular failure had more frequent hypercapnia [54 (39–71) versus 38 (29–47) mmHg, p = 0.002] (Additional file 1: Table S2). In the overall population with SH, median TSH, FT4 and FT3 levels were 51.0 [17.5–94.5] mIU/L, 2.7 [0.0–7.5] pmol/L and 1.3 [0.0–2.2] pmol/L, respectively. Of note, thyroid hormone levels, SH etiology, and triggers did not differ between patients with and without hemodynamic impairment (Additional file 1: Table S2).

### In-ICU organ support and specific management

In-ICU therapeutic management of the whole population is reported in Table 2. ICU nonsurvivors required significantly more frequent vasopressors, inotropic support, invasive mechanical ventilation, and renal replacement therapy compared to ICU survivors (p < 0.001). Of note, 9 (11%) patients required isoprenaline or temporary transvenous ventricular pacing, and two received pericardial drainage. Specific hypothyroidism treatment always included thyroid hormone replacement by levothyroxine associated with a loading dose of 300 [175–400] µg in 43% of patients. Times between hospital or ICU admission to levothyroxine were not associated with ICU survival. Finally, corticosteroids were associated with thyroid hormone replacement in 52 (63%) patients.

**Table 2:** In-ICU complications and management according to ICU survival status

Characteristic	Total	ICU survivors	ICU nonsurvivors	*p*
	(n = 82)	(n = 61)	(n = 21)	
In-ICU complications				
VAP	26 (32)	15 (25)	11 (52)	0.018
Cardiogenic shock	15 (18)	8 (13)	7 (33)	0.052
Acute coronary syndrome	1 (1)	0 (0)	1 (5)	0.256
Cardiac arrest	2 (2)	1 (2)	1 (5)	0.449
Ventricular fibrillation or tachycardia	2 (2)	2 (3)	0 (0)	1.000
Supraventricular tachycardia	3 (4)	2 (3)	1 (5)	1.000
Ileus	8 (10)	6 (10)	2 (9)	1.000
Therapeutic management				
Organ support				
Any vasopressors	30 (37)	18 (29)	12 (57)	0.035
Dobutamine	14 (17)	7 (11)	7 (33)	0.039
Isoprenaline	7 (8)	6 (10)	1 (5)	0.671
Temporary transvenous ventricular pacing	2 (2)	1 (2)	1 (5)	0.449
Non-invasive mechanical ventilation	24 (29)	18 (29)	6 (29)	1.000
Invasive mechanical ventilation	54 (66)	36 (59)	18 (86)	0.033
Duration (days)	6.5 (4.0–12.0)	5.0 (3.0–8.0)	11.0 (5.0–27.0)	0.034
RRT	17 (21)	6 (10)	11 (52)	< 0.001
Pericardial drainage	2 (2)	2 (3)	0 (0)	1.000
Specific hypothyroidism treatment				
Loading dose (binary)	35 (43)	25 (41)	10 (48)	0.618
Loading dose (µg)	300 (175–400)	300 (200–400)	250 (125–375)	0.52
Time between hospital admission and levothyroxine start (days)	1.0 (0.0–4.0)	1.0 (0.0–4.0)	1.0 (1.0–4.0)	0.526
Time between ICU admission and levothyroxine start (days)	0.0 (0.0–2.0)	0.0 (0.0–2.0)	0.0 (− 1.0 to 2.0)	0.919
Starting dose of Levothyroxine (µg)	100 (50–125)	100 (50–125)	100 (62–125)	0.594
Levothyroxine administration				1.000
Intravenous	39 (48)	29 (48)	10 (48)	
Oral	40 (49)	30 (49)	10 (48)	
Corticosteroids	52 (63)	37 (61)	15 (71)	0.440

*ICU* intensive care unit, *VAP* ventilator-associated pneumonia, *RRT* renal replacement therapy

### Patients outcomes

**Fig. 2:**
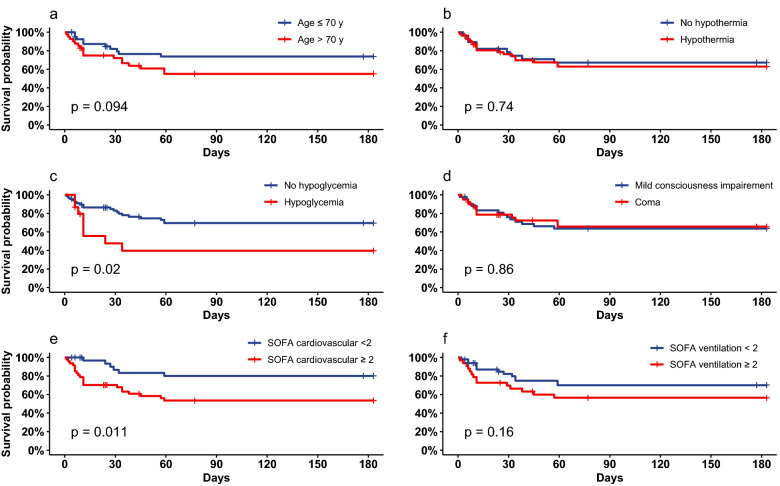
Six-month survival according to **A** age, **B** hypothermia, **C** hypoglycemia, **D** coma, **E** cardiovascular component of the SOFA score, and **F** ventilation component of the SOFA score

ICU mortality was 26% among critically-ill patients with SH. Among 72 patients for whom 6-month survival status was available, mortality reached 39%. ICU nonsurvivors were significantly older [77 (70–81) vs 68 (58–76) years, p = 0.015] and had higher SAPS II and SOFA scores. Moreover, patients with aspiration pneumonia and hemodynamic failure [OR = 10.9 (2.3–104.8) p < 0.001] had a lower ICU survival. Similarly, ICU nonsurvivors had significantly lower hemoglobin levels and higher arterial lactate (Additional file 1: Table S3). During the ICU stay, cardiogenic shock was reported more frequently in nonsurvivors, with a subsequently higher dose of vasopressors and dobutamine (Fig. 2).

### Factors associated with in-ICU and 6-month mortality

After entering age, hemodynamic and ventilation components of the SOFA score, and hypoglycemia in the logistic regression model, age > 70 years [OR 6.01 (1.75–24.1), p = 0.007], hemodynamic failure [OR 11.1 (2.47–84.2), p = 0.005] and ventilation impairment [OR 4.52 (1.27–18.6), p = 0.025] were independently associated with in-ICU mortality (Table 3). Similarly, the logistic regression model with 6-month survival as the dependent variable yielded the same results (Additional file 1: Table S4).

**Table 3:** Predictive factors associated with ICU mortality in critically-ill adults with severe hypothyroidism

	Univariate analysis			Multivariate model		
Characteristic	OR^a^	95% CI^b^	*p*	Adjusted OR^a^	95% CI^b^	*p*
Age > 70 years	3.31	1.04–11.92	0.041	6.01	1.75–24.1	0.007
SOFA cardiovascular ≥ 2	10.90	2.31–104.78	< 0.001	11.1	2.47–84.2	0.005
SOFA ventilation ≥ 2	3.28	1.06–10.77	0.023	4.52	1.27–18.6	0.025
Hypoglycemia	2.28	0.57–8.63	0.19	4.25	0.94–21.2	0.064

^a^*OR*  odds ratio

^b^*CI*  confidence interval

## Discussion

Herein, we report the largest multicenter cohort of SH patients admitted to ICU. SH is an extremely rare thyroid emergency, associated with a significant in-ICU and 6-month mortality of 26% and 39%, respectively. Though clinical presentation may encompass multiple known signs of hypothyroidism, SH-related hemodynamic and respiratory failures at ICU admission were strongly associated with a higher likelihood of mortality. Thyroid hormone replacement (levothyroxine) was consistently provided, although the route of administration and loading dose varied. Moreover, this treatment was frequently combined with steroids.

Thyroiditis and thyroidectomy were the two main identified causes of hypothyroidism leading to SH. Autoimmune thyroiditis is considered the first etiology of hypothyroidism in iodine-sufficient worldwide areas which affects preferentially middle-life women [1, 19]. Indeed, a 3:1 sex ratio favoring women and an age of 70 (59–78) years were reported in our population. A history of levothyroxine discontinuation might also be associated with worse clinical manifestations [12]. In addition, amiodarone was an important triggering factor of SH in our population which reinforces the important association between this drug with high iodine content and critically ill thyroid diseases, including SH or thyroid storm [20, 21]. Hence, while amiodarone-induced hypothyroidism is frequent [22] and does not necessarily require amiodarone discontinuation [23], thyroid function should be monitored regularly after amiodarone prescription [23, 24].

SH should be recognized promptly to initiate thyroid replacement, monitoring, and treatment of potentially related life-threatening organ failures. Developing reliable diagnosis scores may help to identify earlier patients with SH. Indeed, our results externally validate the performance of the diagnosis scoring system of myxedema coma proposed by Popoveniuc et al. [18], as the median score of our population was ≥ 60, which is highly suggestive of myxedema coma. However, this score was not associated with outcomes in our population which precludes using this score as a predictive survival model. Based on our results, combining with that score additional parameters related to hemodynamic and respiratory status at ICU admission could better predict the likely prognosis of this population.

Among clinical manifestations reported in our cohort of SH patients, neurological impairment was ubiquitous with, however, various severity forms, such as coma, somnolence, and seizures. Importantly, coma affected only one-half of our patients which is consistent with findings from a national database Japanese cohort reporting that only one-third of SH patients presented a coma at hospital admission [13]. Myxedema coma, frequently used to define SH, may be misleading as hemodynamic and ventilation failures are also frequent clinical presentations which are, contrary to coma, associated with worse outcomes. The hemodynamic effects of a deficit in thyroid hormone combine with an increase in systemic vascular resistance and a decrease in cardiac contractility leading to a decrease in the cardiac output [25–27]. Severe bradycardia and arrhythmias related to abnormal cardiac repolarization and prolonged QT interval may also occur [28, 29], as well as cardiac tamponade due to the accumulation of fluid rich in mucopolysaccharides in the pericardium [2]. Moreover, hemodynamic instability could be worsened by thyroid hormone introduction in several reports [8]. Several hypotheses are proposed to explain the high incidence of respiratory failure in this population. First, aspiration pneumonia was frequent and could be related to neurological impairment. Second, more than one-third of our patients had bradypnea, and 66% required invasive mechanical ventilation. Impaired ventilatory response to hypoxia and hypercapnia [30], partial obstruction of the upper airway, and neuromuscular and diaphragmatic dysfunction have been already reported in this context and may prolong mechanical ventilation duration [2, 30–32].

For decades, levothyroxine remains the first line treatment for thyroid replacement in SH, whereas triiodothyronine (T_3_) was associated with more side effects and hemodynamic instability in this setting. However, the initial dose and the route of administration are still matters of debate [2] as aggressive levothyroxine replacement at the onset of SH treatment may increase the risk of myocardial infarction or arrhythmias [8]. As illustrated by the large variability in dose, route of administration, and loading dose in our population, thyroid replacement in the context of SH should be pragmatic and adapted to the patient’s age, medical history, and critical illness condition. The current American Thyroid Association (ATA) guidelines recommend intravenous administration of levothyroxine at an initial dose of 200–400 µg with lower doses given for very young or older patients and those with a history of coronary disease or arrhythmia [14]. Moreover, data are still scarce regarding the benefit of combining corticosteroids with thyroid hormone replacement. To date, guidelines advocate its use at the early phase of SH to possibly treat associated autoimmune adrenal insufficiency secondary to pituitary or hypothalamic diseases [14].

Our study’s strengths include the large cohort investigated and characterized in detail, and its multicenter design, with 6-month post-ICU-admission follow-up. However, it has several limitations that should be highlighted. The first is inherent to its retrospective design. Missing follow-up thyroid-hormone dosages precluded analysis of any potential relationship between clinical evolution and thyroid-hormone-level kinetics. In addition, some residual confounding factors might have biased our results. Second, data collection spanned 18 years. Therefore, we cannot rule out that the standard of care for critically ill patients has not changed over the study. However, European and American guidelines regarding the first-line treatment of hypothyroidism did not change during the study period [14, 33]. Third, SH is a rare emergency thyroid disease not clearly defined. Thus, our highly selective inclusion criteria could have restricted the inclusion of critically-ill patients with the most severe form of hypothyroidism with at least one organ failure and might have underestimated the incidence rate of this rare disease. Finally, calcemia at ICU admission was missing for 54/82 patients, which precludes analyzing the respective incidence of associated hypoparathyroidism.

## Conclusion

SH is a rare life-threatening endocrine emergency with various clinical presentations leading to ICU admission. Half of these severe patients have a coma but neurological clinical features could be limited to mild consciousness alteration or seizures. Based on 82 patients with SH admitted to ICUs, overall ICU, and 6-month post-admission mortality rates were 26% and 39%. Older age, hemodynamic and respiratory failure, but not neurological failure were strongly associated with fatal outcomes. This very high mortality for a reversible disease prompts early diagnosis and rapid levothyroxine administration with close cardiac and hemodynamic monitoring. Data are still warranted to better define the appropriate dose and route of administration of this necessary treatment.

## Supplementary Information

**Additional file 1: Figure S1.** Flowchart of patient selection from participating ICUs. **Table S1.** Amount of Missing Data for Each Variable Included in the Analysis. **Table S2.** Characteristics of Severe Hypothyroidism Patients According to the Presence of a Circulatory Failure at ICU Admission. **Table S3.** Clinical and Biological Features at ICU Admission according to ICU survival. **Table S4. **Predictive Patient Factors Associated with 6-month Mortality in Critically ill Adults with Severe Hypothyroidism.

## Data Availability

The data sets used and/or analyzed during the current study are available from the corresponding author on reasonable request.

## Abbreviations

ATA: American Thyroid Association
CI: Confidence interval
FT3: Free triiodothyronine
FT4: Free thyroxine
GCS: Glasgow coma scale
ICU: Intensive care unit
IQR: Interquartile range
LVEF: Left ventricular ejection fraction
MC: Myxedema coma
OR: Odds ratio
SAPS II: Simplified acute physiology score II
SH: Severe hypothyroidism
SOFA: Sequential organ-failure assessment
TSH: Thyroid-stimulating hormone
