# Supplementary material for: Critically ill severe hypothyroidism: a retrospective multicenter cohort study
Source: Ann Intensive Care. 2023 Mar 9;13:15. doi: 10.1186/s13613-023-01112-1 (PMC9998819; doi:10.1186/s13613-023-01112-1)
Supplement: Supplementary file 1 — Additional file 1: Figure S1. Flowchart of patient selection from participating ICUs. Table S1. Amount of Missing Data for Each Variable Included in the Analysis. Table S2. Characteristics of Severe Hypothyroidism Patients According to the Presence of a Circulatory Failure at ICU Admission. Table S3. Clinical and Biological Features at ICU Admission according to ICU survival. Table S4. Predictive Patient Factors Associated with 6-month Mortality in Critically ill Adults with Severe Hypothyroidism. [file 13613_2023_1112_MOESM1_ESM.docx]

**Online Additional file**

**Critically ill Severe Hypothyroidism: A Retrospective Multicenter Cohort Study**

Simon Bourcier, MD, PhD, Maxime Coutrot, MD, Alexis Ferré, MD, Nicolas Van Grunderbeeck, MD, Julien Charpentier, MD, Sami Hraiech, MD, PhD, Elie Azoulay, MD, PhD, Saad Nseir, MD, PhD, Nadia Aissaoui, MD, PhD, Jonathan Messika, MD, PhD, Pierre Fillatre^1^, MD, Romain Persichini, MD, Serge Carreira, MD, Alexandre Lautrette, MD, PhD , Clément Delmas, MD , Nicolas Terzi, MD, PhD, Bruno Mégarbane, MD, PhD, Jean-Baptiste Lascarrou, MD, PhD, Keyvan Razazi, MD, PhD, Xavier Repesssé, MD, Claire Pichereau, MD, Damien Contou, MD, Aurélien Frérou, MD, François Barbier, MD, PhD, Stephan Ehrmann, MD, PhD, Etienne de Montmollin, MD, PhD, Benjamin Sztrymf, MD, Elise Morawiec, MD, Naïke Bigé, MD, PhD, Danielle Reuter, MD, David Schnell, MD, Olivier Ellrodt, MD, Jean Dellamonica, MD, PhD, Alain Combes, MD, PhD, Matthieu Schmidt, MD, PhD

**Figure S1.** Flowchart of patient selection from participating ICUs

**Table S1.** Amount of Missing Data for Each Variable Included in the Analysis.

**Table S2.** Characteristics of Severe Hypothyroidism Patients According to the Presence of a Circulatory Failure at ICU Admission

**Table S3.** Clinical and Biological Features at ICU Admission according to ICU survival

**Table S4.** Predictive Patient Factors Associated with 6-month Mortality in Critically ill Adults with Severe Hypothyroidism.

**Figure S1.** Flowchart of patient selection from participating ICUs

**Table S1. Amount of Missing Data for Each Variable Included in the Analysis**

| **Variables** | **Available data, N (%)** |
| --- | --- |
| **At ICU admission** |  |
| Age (years) | 82 (100.0) |
| Sex Female | 82 (100.0) |
| SAPS II | 82 (100.0) |
| Charlson score | 82 (100.0) |
| SOFA | 82 (100.0) |
| SOFA cardiovascular | 82 (100.0) |
| SOFA respiratory | 82 (100.0) |
| SOFA renal | 82 (100.0) |
| SOFA liver | 81 (98.8) |
| SOFA coagulation | 82 (100.0) |
| SOFA CNS | 82 (100.0) |
| De novo hypothyroidism | 82 (100.0) |
| **Clinical medical history** |  |
| Weight gain | 82 (100.0) |
| Temperature < 35°C | 82 (100.0) |
| Bradypnea | 82 (100.0) |
| Somnolence (9≤Glasgow≤14) | 82 (100.0) |
| Coma (Glasgow<9) | 82 (100.0) |
| Seizures | 82 (100.0) |
| Hypoglycemia | 82 (100.0) |
| Skin oedema | 82 (100.0) |
| Goiter | 82 (100.0) |
| Macroglossia | 82 (100.0) |
| Ileus | 82 (100.0) |
| Aspiration pneumonia | 82 (100.0) |
| Heart rate (beats / min) | 75 (91.5) |
| Heart rate < 50/ min | 82 (100.0) |
| Supraventricular tachycardia | 82 (100.0) |
| Ventricular fibrillation or tachycardia | 82 (100.0) |
| Cardiac arrest before ICU | 82 (100.0) |
| Non specific ST-T changes | 82 (100.0) |
| Bundle branch blocks | 82 (100.0) |
| TSH (mIU/L) | 78 (95.1) |
| FT3 (pmol/L) | 47 (57.3) |
| FT4 (pmol/L) | 62 (75.6) |
| **First 24-hr in-ICU parameter** |  |
| Heart rate (beats / min) | 79 (96.3) |
| Heart rate < 50/ min | 82 (100.0) |
| SBP (mmHg) | 79 (96.3) |
| SDP (mmHg) | 78 (95.1) |
| Lowest temperature (°C) | 76 (92.7) |
| Glasgow | 81 (98.8) |
| **First 24-hr in-ICU biological finding** |  |
| Sodium (mmol/L) | 79 (96.3) |
| Creatinine phosphokinase (IU/L) | 65 (79.3) |
| Total bilirubin (µmol/L) | 68 (82.9) |
| Aspartate transaminase (IU/L) | 73 (89.0) |
| Alanine transaminase (IU/L) | 72 (87.8) |
| Prothrombin time (%) | 70 (85.4) |
| Creatinine (µmol/L) | 75 (91.5) |
| Urea (mmol/L) | 77 (93.9) |
| Hemoglobin (g/dL) | 77 (93.9) |
| Leukocyte count (G/L) | 77 (93.9) |
| Platelets (G/L) | 75 (91.5) |
| Arterial lactate (mmol/L) | 66 (80.5) |
| Arterial pH | 79 (96.3) |
| PCO2, mmHg | 79 (96.3) |
| PO2, mmHg | 78 (95.1) |
| HCO_3_ (mmol/L) | 79 (96.3) |
| Glycemia (mmol/L) | 66 (80.5) |
| **TTE findings** |  |
| Systolic left ventricular dysfunction | 57 (69.5) |
| LVEF (%) | 41 (50.0) |
| Left ventricular dilation | 43 (52.4) |
| Pericardial effusion | 49 (59.8) |
| **In-ICU complications** | 82 (100.0) |
| **Therapeutic management** |  |
| **Organ support** |  |
| Vasopressors | 82 (100.0) |
| Dobutamine | 82 (100.0) |
| Isoprenaline | 82 (100.0) |
| SEES | 82 (100.0) |
| Noninvasive mechanical ventilation | 82 (100.0) |
| Invasive mechanical ventilation | 82 (100.0) |
| Duration | 54 (65.9) |
| RRT | 81 (98.8) |
| **Pericardial drainage** | 82 (100.0) |
| **Specific hypothyroidism treatment** |  |
| Loading dose (binary) | 82 (100.0) |
| Loading dose (µg) | 82 (100.0) |
| Time between hospital admission and levothyroxine start | 81 (98.8) |
| Time between ICU admission and levothyroxine start | 81 (98.8) |
| Starting dose of Levothyroxine | 80 (97.6) |
| Levothyroxine route | 82 (100.0) |
| Corticosteroids | 82 (100.0) |
| **6-months vital status** | 72 (87.8) |

**Table S2. Characteristics of Severe Hypothyroidism Patients According to the Presence of a Circulatory Failure at ICU Admission**

| **Characteristic** | **Total** | **No hemodynamic impairment** | **Hemodynamic**  **impairment** | ***p*** |
| --- | --- | --- | --- | --- |
| **At ICU admission** | **N=82** | **N=35** | **N=47** |  |
| **Age (years)** | 70 (59 – 78) | 72 (61 – 76) | 70 (55 – 79) | 0.629 |
| **Sex female** | 61 (74.4) | 27 (77.1) | 34 (72.3) | 0.799 |
| **SOFA score** | 8 (6 – 12) | 6 (3 – 7) | 11 (8 – 13) | <0.001 |
| Cardiovascular component | 3 (0 – 4) | 0 (0 – 1) | 4 (4 – 4) | <0.001 |
| Ventilation component | 1 (0 – 2) | 1 (0 – 2) | 2 (1 – 3) | 0.016 |
| Renal component | 0 (0 – 2) | 0 (0 – 1) | 1 (0 – 3) | 0.005 |
| Liver component | 0 (0 – 0) | 0 (0 – 0) | 0 (0 – 0) | 0.624 |
| Coagulation component | 0 (0 – 2) | 0 (0 – 1) | 0 (0 – 2) | 0.283 |
| Neurological component | 3 (2 – 4) | 3 (2 – 4) | 3 (1 – 4) | 0.930 |
| **De novo hypothyroidism** | 44 (54) | 21 (60) | 23 (49) | 0.375 |
| **Medical history** |  |  |  |  |
| Weight gain | 20 (24) | 9 (26) | 11 (23) | 1.000 |
| Bradypnea | 32 (39) | 18 (51) | 14 (30) | 0.067 |
| Somnolence (9≤Glasgow≤14) | 38 (46) | 17 (49) | 21 (45) | 0.824 |
| Coma | 43 (52) | 20 (57) | 23 (49) | 0.508 |
| Seizures | 10 (12) | 7 (20) | 3 (6) | 0.089 |
| Hypoglycemia | 15 (18) | 6 (17) | 9 (19) | 1.000 |
| Skin oedema | 33 (40) | 12 (34) | 21 (44) | 0.372 |
| Goiter | 6 (7) | 2 (6) | 4 (8) | 1.000 |
| Macroglossia | 12 (15) | 5 (14) | 7 (15) | 1.000 |
| Ileus | 17 (21) | 6 (17) | 11 (23) | 0.587 |
| Aspiration pneumonia | 39 (48) | 12 (34) | 27 (57) | 0.046 |
| Heart rate (beats/min) | 50 (35 – 64) | 55 (44 – 64) | 40 (30 – 63) | 0.050 |
| Heart rate < 50/ min | 54 (66) | 24 (69) | 30 (64) | 0.814 |
| Supraventricular tachycardia | 9 (11) | 1 (3) | 8 (17) | 0.071 |
| Ventricular fibrillation or tachycardia | 1 (1) | 0 (0) | 1 (2) | 1.000 |
| Cardiac arrest before ICU | 8 (10) | 0 (0) | 8 (17) | 0.009 |
| Nonspecific ST-T changes | 14 (17) | 7 (20) | 7 (15) | 0.567 |
| Bundle branch blocks | 16 (19) | 5 (14) | 11 (23) | 0.402 |
| TSH (mIU/L) | 51.0 (17.5 – 94.5) | 42.5 (17.0 – 91.5) | 59.0 (21.1 – 94.5) | 0.499 |
| FT3 (pmol/L) | 1.3 (0.0 – 2.2) | 1.5 (0.9 – 2.6) | 0.9 (0.0 – 2.1) | 0.150 |
| FT4 (pmol/L) | 2.7 (0.0 – 7.5) | 3.0 (0.0 – 7.1) | 2.0 (0.0 – 7.9) | 0.983 |
| **First 24-hr in-ICU parameter** |  |  |  |  |
| Heart rate (beats/min) | 55 (41 – 70) | 60 (45 – 70) | 51 (40 – 70) | 0.344 |
| Heart rate < 50/ min | 36 (44) | 14 (40) | 22 (47) | 0.654 |
| SBP (mmHg) | 100 (84 – 126) | 105 (89 – 132) | 99 (80 – 114) | 0.084 |
| DBP (mmHg) | 60 (52 – 72) | 64 (56 – 77) | 57 (50 – 70) | 0.048 |
| Lowest temperature (°C) | 34.1 (31.1 – 35.9) | 34.1 (32.1 – 35.8) | 34.2 (30.2 – 35.9) | 0.477 |
| Glasgow scale | 8 (5 – 12) | 8 (5 – 11) | 7 (4 – 13) | 0.715 |
| **First 24-hr in-ICU biological finding** |  |  |  |  |
| Sodium (mmol/L) | 139 (132 – 144) | 139 (135 – 144) | 136 (132 – 145) | 0.455 |
| Creatinine phosphokinase (IU/L) | 258 (130 – 1221) | 235 (134 – 644) | 285 (130 - 1377) | 0.542 |
| Total bilirubin (µmol/L) | 9.0 (5.2 - 13.5) | 9.5 (6.8 - 15.0) | 9.0 (5.0 - 11.5) | 0.574 |
| Aspartate transaminase (IU/L) | 74 (34 – 146) | 59 (36 – 123) | 77 (32 – 158) | 0.724 |
| Alanine transaminase (IU/L) | 49 (29 – 91) | 44 (26 – 89) | 50 (30 – 92) | 0.615 |
| Prothrombin time (%) | 75 (60 – 90) | 84.0 (69 – 96) | 71 (40 – 80) | 0.003 |
| Creatinine (µmol/L) | 97 (69 – 153) | 80 (61 – 102) | 109 (76 – 213) | 0.005 |
| Urea (mmol/L) | 8.9 (5.3 – 15.8) | 7.2 (5.3 – 13.8) | 9.4 (6.0 – 20.4) | 0.177 |
| Hemoglobin (g/dL) | 10.5 (9.4 – 12.0) | 11.5 (10.1 – 12.2) | 10.0 (8.9 – 11.3) | 0.014 |
| Leukocyte count (G/L) | 7.1 (3.8 – 12.1) | 6.1 (3.5 – 9.2) | 8.9 (4.4 – 14.9) | 0.048 |
| Platelets (G/L) | 152 (81 – 196) | 158 (94 – 218) | 144 (81 – 182) | 0.381 |
| Arterial lactate (mmol/L) | 1.7 (1.1 – 3.3) | 1.2 (0.9 – 2.5) | 2.2 (1.5 – 4.1) | 0.014 |
| Arterial pH | 7.4 (7.2 – 7.4) | 7.4 (7.2 – 7.4) | 7.3 (7.2 – 7.4) | 0.669 |
| PCO2, mmHg | 41.0 (32.5 – 59.0) | 54.0 (39.5 – 71.0) | 38.0 (29.2 – 47.5) | 0.002 |
| PO2, mmHg | 80.1 (63.2 – 119.0) | 77.0 (61.0 – 94.8) | 84.0 (65.0 – 151.0) | 0.113 |
| HCO_3_ (mmol/L) | 23.0 (18.5 – 29.0) | 27.0 (23.0 – 32.0) | 20.0 (17.0 – 25.0) | <0.001 |
| Glycemia | 4.9 (3.5 – 8.0) | 4.9 (4.0 – 5.9) | 4.9 (3.4 – 8.6) | 0.990 |
| **TTE findings** |  |  |  |  |
| Left ventricular systolic dysfunction | 30 (37) | 6 (17) | 24 (51) | 0.086 |
| LVEF (%) | 45 (35 – 60) | 60 (47 – 60) | 40 (35 – 57) | 0.044 |
| Left ventricular dilation | 8 (10) | 2 (6) | 6 (13) | 1.000 |
| Pericardial effusion | 20 (24) | 6 (17) | 14 (30) | 1.000 |
| **In-ICU complications** |  |  |  |  |
| VAP | 26 (32) | 8 (23) | 18 (38) | 0.137 |
| Acute coronary syndrome | 1 (1) | 0 (0) | 1 (2) | 1.000 |
| Cardiogenic shock | 15 (18) | 1 (3) | 14 (30) | 0.001 |
| Cardiac arrest | 2 (2) | 0 (0) | 2 (4) | 0.505 |
| Ventricular fibrillation or tachycardia | 2 (2) | 0 (0) | 2 (4) | 0.505 |
| Supraventricular tachycardia | 3 (3.7) | 0 (0.0) | 3 (6.4) | 0.257 |
| Ileus | 8 (9.8) | 6 (17.1) | 2 (4.3) | 0.068 |
| **Therapeutic management** |  |  |  |  |
| **Organ support** |  |  |  |  |
| Vasopressors | 30 (37) | 2 (6) | 28 (60) | <0.001 |
| Dobutamine | 14 (17) | 1 (3) | 13 (28) | 0.003 |
| Isoprenaline | 7 (8) | 1 (3) | 6 (13) | 0.230 |
| Temporary transvenous ventricular pacing | 2 (2) | 0 (0) | 2 (4) | 0.505 |
| Noninvasive mechanical ventilation | 24 (29) | 13 (37) | 11 (23) | 0.222 |
| Invasive mechanical ventilation | 54 (66) | 19 (54) | 35 (74) | 0.065 |
| Duration (days) | 6.5 (4.0 – 12.0) | 5.0 (3.0 – 7.5) | 7.0 (4.5 – 21.0) | 0.108 |
| RRT | 17 (21) | 2 (6) | 15 (32) | 0.005 |
| Pericardial drainage | 2 (2) | 1 (3) | 1 (2) | 1.000 |
| **Specific hypothyroidism treatment** |  |  |  |  |
| Loading dose (binary) | 35 (43) | 13 (37) | 22 (47) | 0.499 |
| Loading dose (µg) | 300 (175–400) | 300 (200–400) | 300 (131–375) | 0.29 |
| Time between hospital admission and levothyroxine start | 1.0 (0.0 – 4.0) | 2.0 (0.0 – 4.5) | 1.0 (0.0 – 2.0) | 0.150 |
| Time between ICU admission and levothyroxine start | 0.0 (0.0 – 2.0) | 0.0 (0.0 – 2.0) | 0.0 (0.0 – 1.0) | 0.332 |
| Starting dose of Levothyroxine | 100 (50 – 125) | 100 (50 – 112) | 100 (50 – 125) | 0.819 |
| IV | 39 (48) | 18 (51) | 21 (45) |  |
| Oral | 40 (49) | 16 (46) | 24 (51) |  |
| Corticosteroids | 52 (63) | 22 (63) | 30 (64) | 1.000 |
| **Etiology**  **Primary** | 76 (93) | 32 (91) | 44 (94) | 0.629 |
| Throiditis | 24 (29) | 10 (29) | 14 (30) |  |
| Thyroidectomy | 16 (19) | 4 (11) | 12 (25) |  |
| Unknown | 20 (24) | 9 (26) | 11 (23) |  |
| Graves’ disease | 1 (1) |  | 1 (2.1) |  |
| Atrophic thyroiditis | 1 (1) | 1 (3) |  |  |
| Congenitale | 1 (1) |  | 1 (2) |  |
| **Central** | 6 (7) | 3 (9) | 3 (6) |  |
| **Trigger** |  |  |  | 0.717 |
| Levothyroxine discontinuation | 23 (28) | 8 (23) | 15 (32) |  |
| Sepsis | 12 (15) | 7 (20) | 5 (11) |  |
| Amiodarone | 9 (11) | 3 (9) | 6 (13) |  |
| Drugs | 5 (6) | 2 (6) | 3 (6) |  |
| None | 33 (40) | 15 (43) | 18 (38) |  |
| **Outcome** |  |  |  |  |
| ICU survival | 61 (74) | 33 (94) | 28 (60) | <0.001 |
| 6-months survival | 44 (54) | 24 (69) | 20 (43) | 0.007 |
| 6-months status Missing | 10 (12) | 5 (14) | 5 (11) |  |

ICU: intensive care unit, SBP: systolic blood pressure, DBP: diastolic blood pressure, TTE: transthoracic echocardiography, LVEF: left ventricular ejection fraction, VAP: ventilator-associated pneumonia, RRT: renal replacement therapy

**Table S3. Clinical and biological features at ICU admission according to ICU survival**

| **Characteristic** | **Total** | **ICU survivors** | **ICU nonsurvivors** | ***p*** |
| --- | --- | --- | --- | --- |
|  | **(n=82)** | **(n=61)** | **(n=21)** |  |
| **First 24-hr in-ICU parameter** |  |  |  |  |
| Heart rate (beats/min) | 55 (41 – 70) | 55 (40 – 65) | 68 (45 – 76) | 0.130 |
| Heart rate < 50/ min | 36 (43.9) | 28 (45.9) | 8 (38.1) | 0.615 |
| SBP (mmHg) | 100 (84 – 126) | 101 (84 – 126) | 98 (80 – 116) | 0.722 |
| DBP (mmHg) | 60 (52 – 72) | 61 (51 – 71) | 60 (56 – 71) | 0.608 |
| Lowest temperature (°C) | 34.1 (31.1 – 35.9) | 34.0 (31.6 – 35.6) | 35.2 (30.2 – 36.0) | 0.389 |
| Glasgow | 8 (5 – 12) | 8 (6 – 12) | 7 (3 – 12) | 0.289 |
| **First 24-hr in-ICU biological finding** |  |  |  |  |
| Sodium (mmol/L) | 139 (132 – 144) | 139 (132 – 143) | 139 (133 – 146) | 0.627 |
| Creatinine phosphokinase (IU/L) | 258 (130 – 1221) | 222 (129 – 1008) | 723 (200 – 1454) | 0.146 |
| Total bilirubin (µmol/L) | 9.0 (5.2 – 13.5) | 9.0 (5.0 – 15.0) | 9.0 (6.0 – 11.5) | 0.900 |
| Aspartate transaminase (IU/L) | 74 (34 – 146) | 56 (34 – 127) | 95 (49 – 225) | 0.254 |
| Alanine transaminase (IU/L) | 49 (29 – 91) | 49 (29 – 88) | 53 (30 – 110) | 0.494 |
| Prothrombin time (%) | 75 (60 – 90) | 76 (66 – 92) | 51 (22 – 82) | 0.064 |
| Creatinine (µmol/L) | 97 (69 – 153) | 86 (63 – 149) | 106 (97 – 255) | 0.050 |
| Urea (mmol/L) | 8.9 (5.3 – 15.8) | 8.2 (5.3 – 15.0) | 9.4 (6.7 – 18.7) | 0.589 |
| Hemoglobin (g/dL) | 10.5 (9.4 – 12.0) | 11.0 (9.8 – 12.1) | 9.5 (8.9 – 10.6) | 0.032 |
| Leukocyte count (G/L) | 7.1 (3.8 – 12.1) | 6.7 (3.5 – 10.6) | 8.2 (5.5 – 14.3) | 0.131 |
| Platelets (G/L) | 152 (81 – 196) | 167 (99 – 219) | 107 (70 – 160) | 0.091 |
| Arterial lactate (mmol/L) | 1.7 (1.1 – 3.3) | 1.6 (1.0 – 3.0) | 3.0 (1.6 – 6.2) | 0.012 |
| Arterial pH | 7.4 (7.2 – 7.4) | 7.4 (7.2 – 7.4) | 7.3 (7.2 – 7.4) | 0.659 |
| PCO2, mmHg | 41.0 (32.5 – 59.0) | 41.0 (33.0 – 61.5) | 41.3 (30.0 – 52.0) | 0.520 |
| PO2, mmHg | 80.1 (63.2 – 119.0) | 86.0 (63.5 – 127.5) | 68.0 (62.5 – 85.5) | 0.157 |
| HCO_3_ (mmol/L) | 23.0 (18.5 – 29.0) | 24.9 (19.0 – 30.0) | 21.0 (18.0 – 25.0) | 0.188 |
| Glycemia (mmol/L) | 4.9 (3.5 – 8.0) | 4.9 (4.0 – 8.9) | 4.6 (3.0 – 7.2) | 0.491 |
| **TTE findings** |  |  |  |  |
| Systolic left ventricular dysfunction | 30 (37) | 15 (25) | 15 (71) | 0.006 |
| LVEF (%) | 45 (35 – 60) | 50 (40 – 60) | 35 (24 – 41) | 0.004 |
| Left ventricular dilation | 8 (10) | 4 (7) | 4 (19.) | 0.404 |
| Pericardial effusion | 20 (24) | 15 (24.6) | 5 (24) | 0.754 |
| **6-months vital status alive** | 44 (54) | 44 (72) | 0 (0) | <0.001 |
| Missing status | 10 (12) | 10 (16) | 0 (0) |  |

ICU: intensive care unit, SBP: systolic blood pressure, DBP: diastolic blood pressure, TTE: transthoracic echocardiography, LVEF: left ventricular ejection fraction, VAP: ventilator-associated pneumonia, RRT: renal replacement therapy..

**Table S4. Predictive Patient Factors Associated with 6-month mortality in critically ill adults with severe hypothyroidism.**

|  | **Multivariate model** | | |
| --- | --- | --- | --- |
| **Characteristic** | **Adjusted OR***^1^* | **95% CI***^2^* | **p-value** |
| **Age > 70 years** | 3.87 | 1.28 – 13.20 | 0.021 |
| **SOFA cardiovascular ≥ 2** | 3.57 | 1.14 – 12.40 | 0.034 |
| **SOFA ventilation** **≥ 2** | 2.59 | 0.82 – 8.83 | 0.110 |
| **Hypoglycemia** | 4.31 | 1.07 – 19.90 | 0.046 |

*^1^*OR = Odds Ratio, ^2^CI = Confidence Interval
